# Supplementary material for: Bacterial Community Diversity and Bacterial Interaction Network in Eight Mosquito Species
Source: Genes (Basel). 2022 Nov 7;13(11):2052. doi: 10.3390/genes13112052 (PMC9690548; doi:10.3390/genes13112052)
Supplement: Supplementary file 1 [file genes-13-02052-s001.zip › genes-1949879-supplementary/Supplementary Material legends.pdf]

Bacterial Community Diversity and Bacterial Interaction Networks in Eight Mosquito Species

Authors: Herculano da Silva, Tatiane M. P. Oliveira, Maria Anice M. Sallum

Departamento de Epidemiologia, Faculdade de Saúde Pública, Universidade de São Paulo, Avenida Doutor Arnaldo 715, São Paulo 01246-904, SP, Brasil

Supplementary Materials

Figure legends

Figure S1: Bar graph depicting the bacterial amplicon sequence variants (ASV) composition at the highest taxonomic level (phylum) for each mosquito sample.

Figure S2: Bar graph depicting the bacterial amplicon sequence variants (ASV) composition at the bottom taxonomic level (genus) for each mosquito sample.

Figure S3: Rarefaction curve. Count of ASVs per given sequencing depth in each mosquito sample. Each line corresponds to a mosquito sample.

Figure S4: The illustration represents ANCOM analysis. Twelve taxa were the most abundant and are marked 1 to 12 from right to left.

Figure S5: Heatmap of bacterial sequences with taxonomic assignment to genus level in each mosquito sample. Each row represents a bacterial taxon and each column corresponds a mosquito species. Abundance data are assigned colours across a gradient from yellow (higher bacterial abundance) to blue (lowest bacterial abundance).

Table legends

Table S1: Count of 16S rRNA raw data and contigs in each mosquito sample.

Table S2: ASVs count in each sample and mosquito species.

Table S3: Shannon index of each mosquito sample.

Table S4: Pairwise permanova results from unweighted and weighted Unifrac distances.

Table S5: ANCOM analysis. Percentile abundances of features by group.

Table S6: Relative abundance of each ASV in each mosquito species.

Table S7: Number of bacterial interactions per mosquito species and number of bacterial taxa related to interactions.
