# Supplementary figures and images for: Bacterial Community Diversity and Bacterial Interaction Network in Eight Mosquito Species
Source: Genes (Basel). 2022 Nov 7;13(11):2052. doi: 10.3390/genes13112052 (PMC9690548; doi:10.3390/genes13112052)

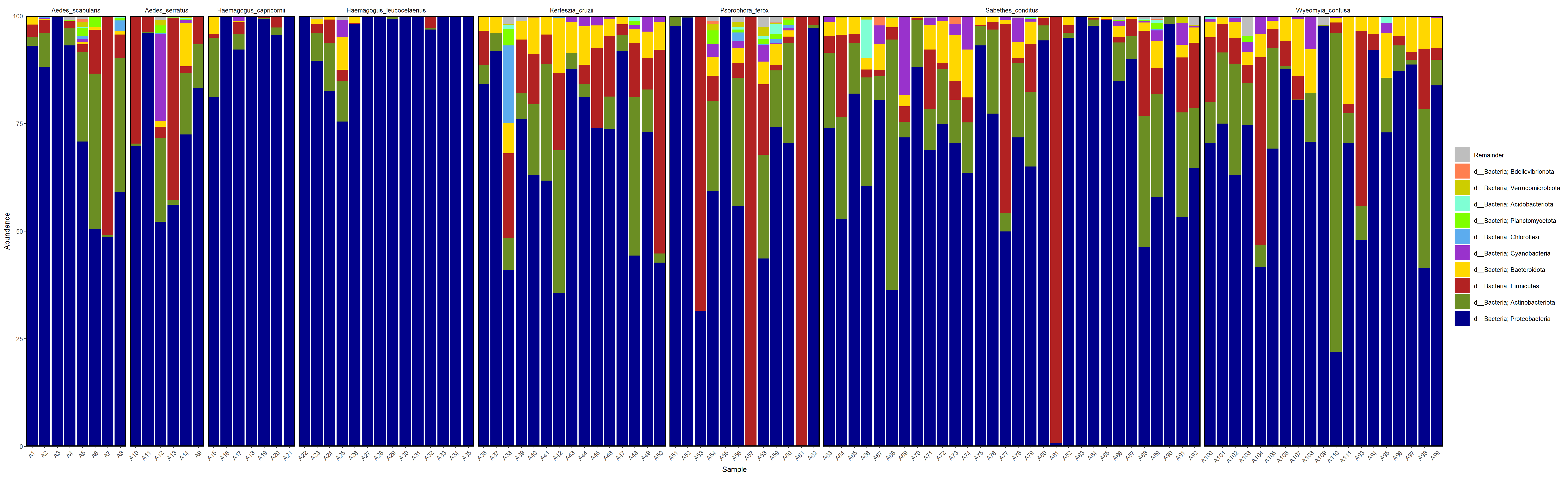

Supplement: Supplementary file 1 [file genes-13-02052-s001.zip › genes-1949879-supplementary/Supplementary Figure S1.png]

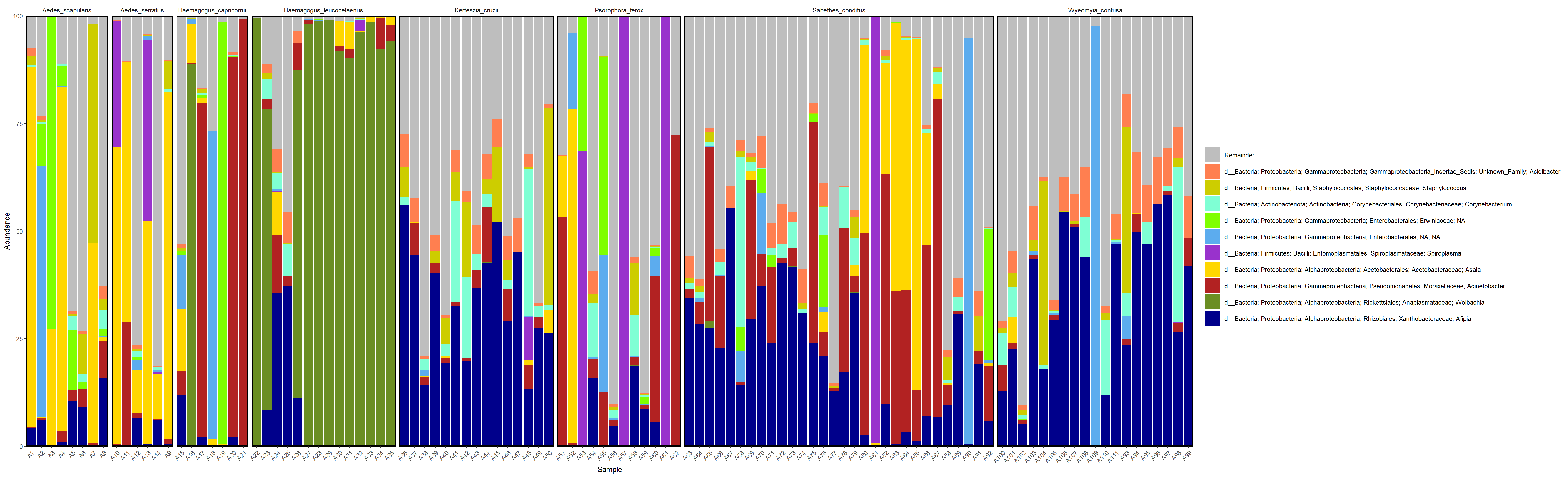

Supplement: Supplementary file 1 [file genes-13-02052-s001.zip › genes-1949879-supplementary/Supplementary Figure S2.png]

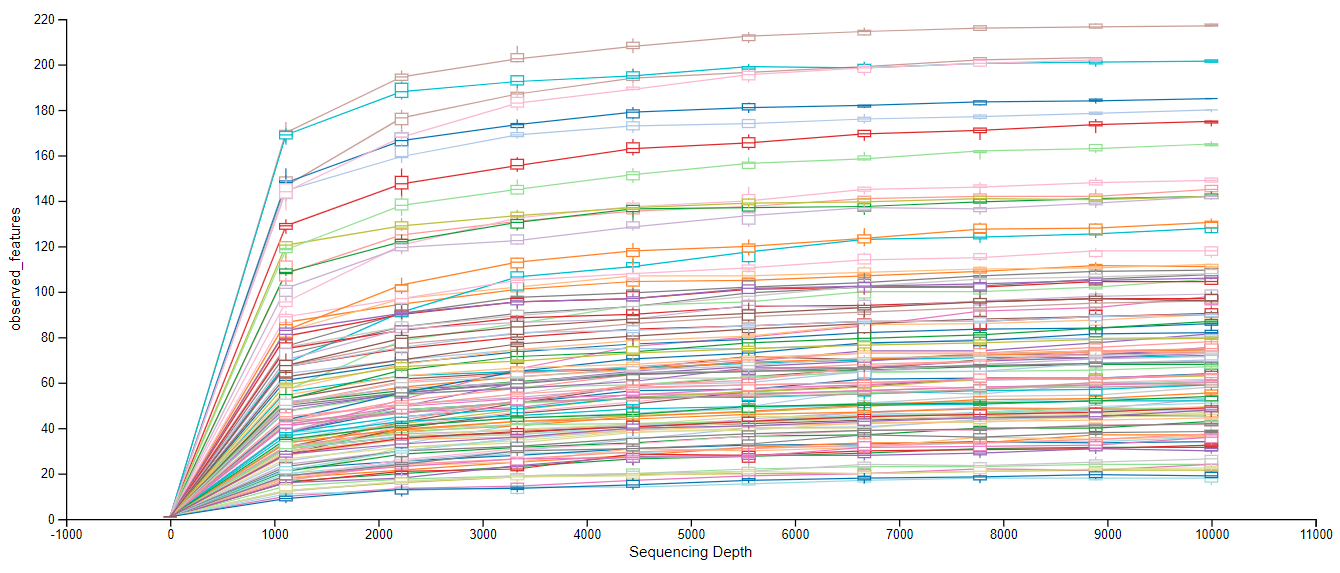

Supplement: Supplementary file 1 [file genes-13-02052-s001.zip › genes-1949879-supplementary/Supplementary Figure S3.tif]

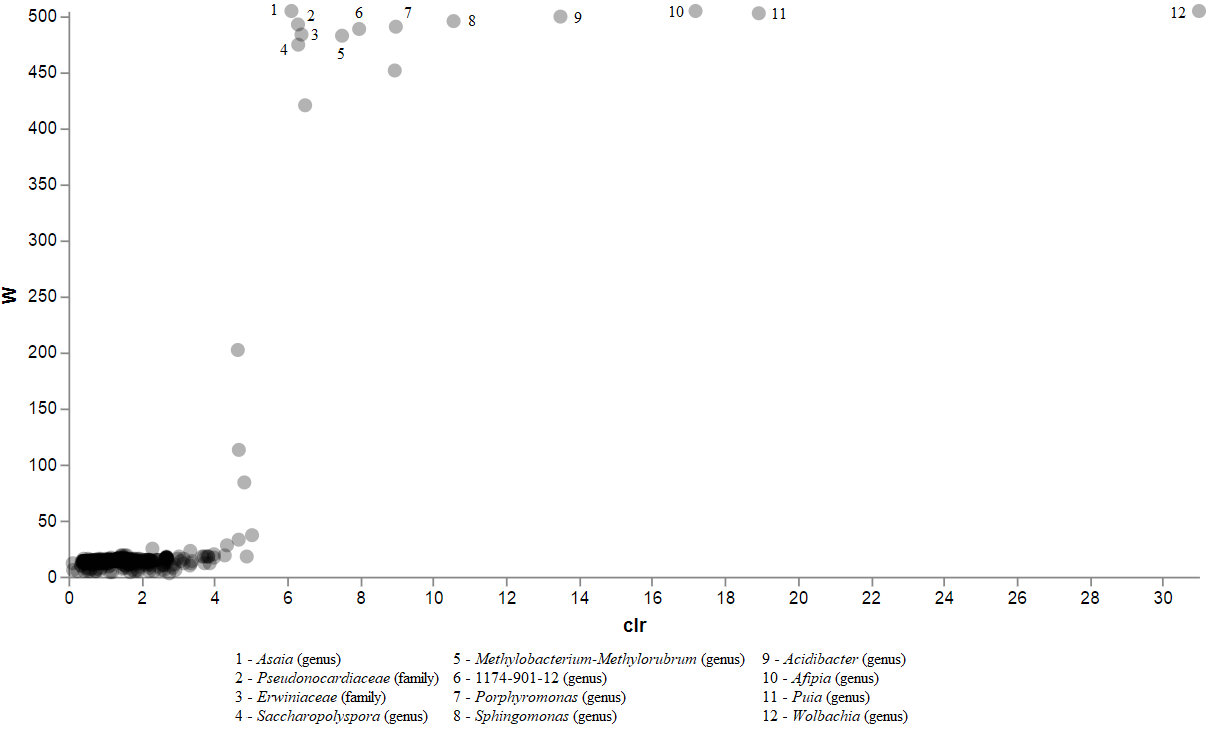

Supplement: Supplementary file 1 [file genes-13-02052-s001.zip › genes-1949879-supplementary/Supplementary Figure S4.tif]

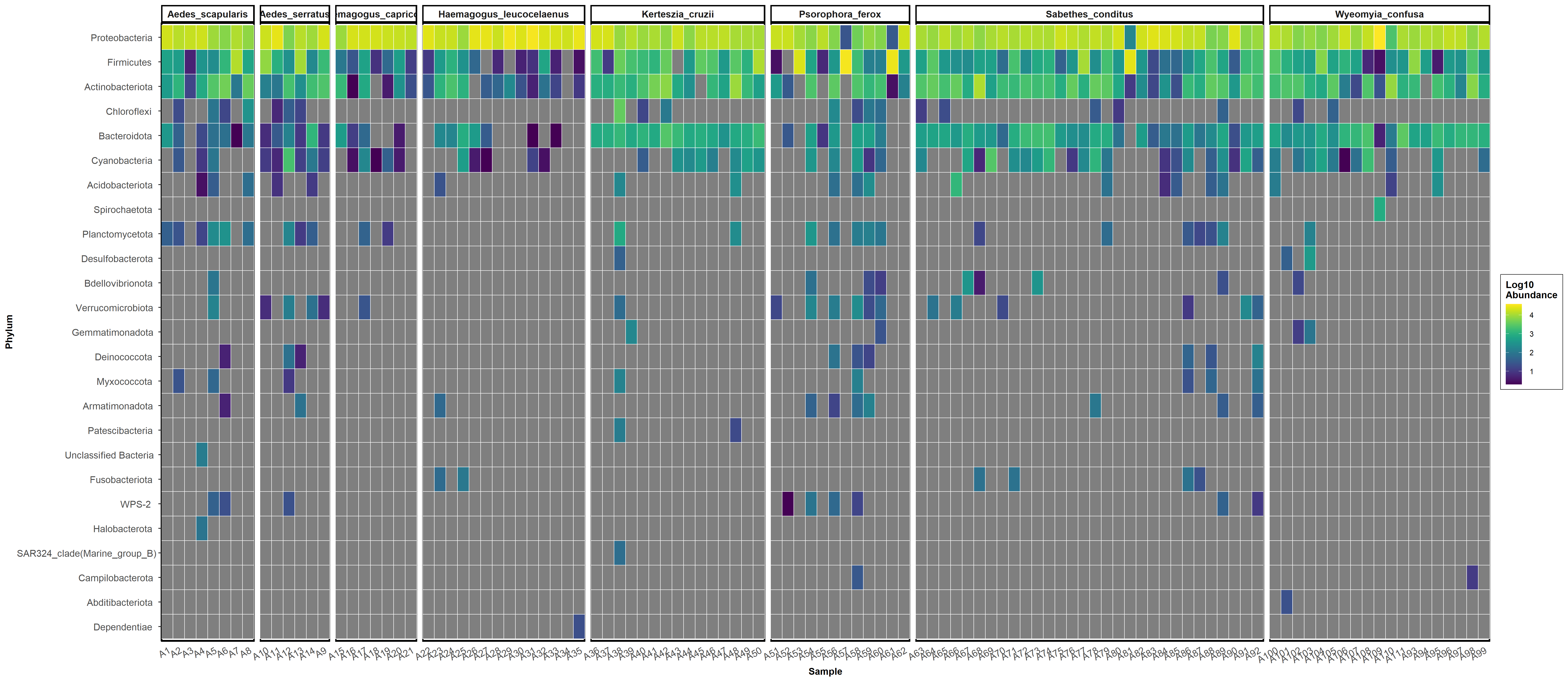

Supplement: Supplementary file 1 [file genes-13-02052-s001.zip › genes-1949879-supplementary/Supplementary Figure S5.png]
